# Recent Advances Review in Plant Extracts-Driven Green Synthesis of Binary-Metal Oxide Nanomaterials for Sustainable Nanotechnology

**DOI:** 10.1155/sci5/2888408

**Published:** 2025-11-28

**Authors:** Nolubabalo Matinise

**Affiliations:** UNESCO-UNISA Africa Chair in Nanosciences-Nanotechnology, College of Graduate Studies, University of South Africa, Muckleneuk Ridge, P.O. Box 392, Pretoria, South Africa

**Keywords:** bimetallic oxides, green synthesis, *Moringa oleifera*, nanomaterials

## Abstract

This review highlights recent advancements in the development of environmentally sustainable and reliable methods for the bio-fabrication of binary metal oxide nanomaterials through plant extract-mediated green methods, with a particular emphasis on *Moringa oleifera.* Known for its rich profile of bioactive compounds, including vitamins, flavonoids, and phenolic acids, it serves as a natural reducing, capping, and chelating agent, facilitating the formation of bimetallic oxide nanostructures (zinc cobalt, zinc iron, and zinc zirconate) through bio-fabrication processes. The plant-derived agents from *M. oleifera* enhance nanomaterial properties, including catalytic activity, stability, and surface area, making them highly suitable for diverse applications in environmental remediation, biomedicine, energy, and sensing technologies. The motivation for this strategy arises from the necessity for eco-friendly, cost-efficient, and scalable techniques that reduce toxicity and eliminate hazardous chemicals. The review elaborates on the mechanisms underlying the formation of bimetallic oxide nanostructures, specifically zinc cobalt (ZnCo_2_O_4_), zinc iron (ZnFe_2_O_4_), and zinc zirconate (ZnZrO_3_), through chemical reactions between salt precursors and bioactive compounds extracted from *M. oleifera* plant natural extract. It emphasizes the principles of green synthesis that align with sustainable nanotechnology, promoting reduced toxicity and cost-effectiveness. This approach addresses the increasing demand for eco-friendly synthetic pathways utilizing plants like *M. oleifera,* microorganisms, and other biological sources, thereby advancing green chemistry and enabling the development of nanomaterials with enhanced functionalities for practical applications.

## 1. Introduction

Nanomaterials, characterized by particle sizes within the 1–100 nm range, have garnered significant scientific interest due to their distinctive physical and chemical properties, such as enhanced thermal conductivity, catalytic activity, nonlinear optical behavior, and high surface area-to-volume ratio [1, 2]. These attributes provide the foundation for their wide-ranging applications in fields including environmental remediation, catalysis, energy storage, sensors, and electronics [2, 3].

Despite their advantages, conventional physical and chemical synthesis methods often pose environmental and health challenges. These approaches typically require high energy inputs, involve toxic chemicals, and generate hazardous waste, raising concerns over sustainability and ecological impact [4–7]. These issues present risks to both health and the environment; there is an urgent need for environmentally benign and sustainable synthesis strategies.

Green synthesis has emerged as a promising alternative, aligning with principles of sustainability and eco-friendliness. Utilizing biological resources such as plant extracts, fungi, and bacteria, these methods enable the production of nanomaterials in a clean, cost-effective, and scalable manner [4]. Among these biological resources, plant-mediated synthesis is particularly attractive due to the abundance of natural reducing agents, including phenolic compounds, flavonoids, vitamins, and phytochemicals that facilitate the reduction of metal ions and stabilize nanoparticles without the use of toxic chemicals or high energy inputs. The natural components of plants serve as both reducing agents, converting metal ions into nanoparticles, and stabilizers, preventing agglomeration and controlling particle growth [8–10].

The advantages of green synthesis extend beyond environmental benefits; they include lower production costs, biocompatibility of the resulting nanomaterials, and suitability for diverse applications such as environmental cleanup, catalysis, and biomedical fields. Recent advances have demonstrated the successful synthesis of various metals and metal oxide nanoparticles, including zinc, cobalt, iron, and zirconate-based nanomaterials, using plant extracts. These eco-friendly nanomaterials show promise for sustainable solutions in environmental and energy-related applications.

This review aims to critically analyze recent advancements in the plant extract-driven green synthesis of binary metal oxides, specifically zinc cobalt (ZnCo_2_O_4_), zinc iron (ZnFe_2_O_4_), and zinc zirconate (ZnZrO_3_). It discusses the underlying mechanisms, characterization techniques, and potential applications of these nanomaterials, as well as the challenges and future directions for integrating green synthesis into sustainable nanotechnology practices.

### 1.1. Fundamentals of Binary-Metal Oxide Nanomaterials

Binary-metal oxide nanomaterials are nanoscale composites made up of two distinct metal oxides, typically ranging in size from 1 to 100 nm, demonstrating exceptional properties that make them ideal for use in catalysis, sensors, energy storage, and environmental remediation. These nanostructures have distinct physicochemical properties that set them apart from their bulk counterparts, such as a high surface-to-volume ratio, increased reactivity, improved catalytic activity, and tunable electronic and optical properties [11–13]. The nanoscale dimension facilitates quantum confinement effects and provides a large number of active sites, increasing their usefulness in a variety of applications [11–14]. The specific properties of these materials are determined by the metal combination, stoichiometric ratios, and morphology (e.g., nanoparticles, nanorods, or nanosheets) as well as their common classification, which includes mixed metal oxide nanoparticles, heterostructure nanomaterials, core-shell, and doped metal oxide nanostructures. Their high stability, low cost, and availability make them especially appealing for environmentally friendly technological applications [15–19].

Among the binary metal oxide various spinel-type oxides recently studied, structures based on bimetallic zinc (ZnM_2_O_4_, where M=Co, Fe, and Zr), such as ZnCo_2_O_4_, ZnO–ZrO_2_, and ZnFe_2_O_4_, are a diverse class of composite nanostructures characterized by their unique combination of physical, chemical, magnetic, and electronic properties, which are largely influenced by their specific crystal structures, such as spinel or heterostructures. These nanomaterials exhibit high electrical conductivity, excellent catalytic activity, magnetic responsiveness, photocatalytic efficiency, and chemical stability [20–22], making them highly suitable for a wide range of applications, including energy storage devices like supercapacitors and lithium-ion batteries, environmental remediation through pollutant degradation, gas sensing, and biomedical applications, such as drug delivery, magnetic resonance imaging (MRI), and cancer treatment [23, 24]. Their structural features, such as surface area, particle size, and crystalline phases, can be tuned to optimize performance in specific applications. Moreover, the synthesis of these nanomaterials is increasingly being achieved through environmentally friendly green methods utilizing plant extracts, which reduce the use of hazardous chemicals and enhance biocompatibility and cost-effectiveness. This approach aligns with sustainable development goals and opens new avenues for large-scale, eco-conscious production of advanced nanomaterials with multifunctional capabilities for industrial, environmental, and healthcare applications. Various researchers have done much biosynthesis of metallic oxides such as zinc, cobalt, and zirconia oxide using various plant extracts, namely fruit extract of *Rosa canina, Aspalathus linearis* natural extract, extracts of *Sageretia thea, Rhamnus virgata* leaf extract, *Aspergillus nidulans*, *Acalypha indica* leaf extract [25–30]. Various plant extracts have been employed as bioreductants and stabilizing agents for the development of bimetallic nanomaterials, includin*g A. linearis* natural extracts, *Moringa Oleifera*, *Dactylopius Coccus, Chrysanthemum* spp. floral waste*, Crataegus monogyna* leaf extract, *Chrysanthemum* spp. flower extract, waste banana peel*, Glycyrrhiza glabra* extract*, Ricinus communis* leaf extract, and *Boswellia carteri* resin [31–40].

### 1.2. The Development of Bimetallic Nanomaterials

**Scheme 1:**
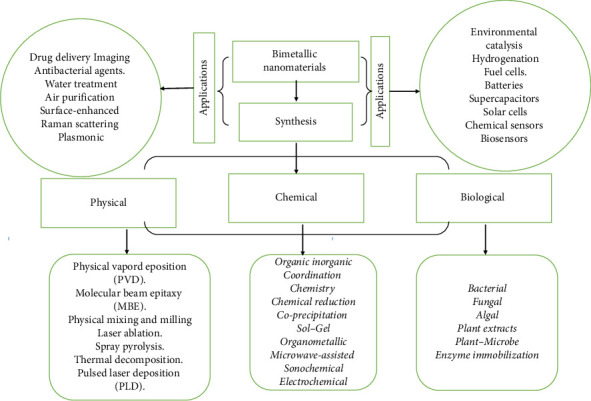
A schematic representation of the synthesis and applications of bimetallic nanomaterials, emphasizing both top–down and bottom–up approaches. The bottom–up approach, which mainly employs chemical and biological sources, involves processes like chemical reduction and green synthesis using biological agents.

Bimetallic oxide nanomaterials have garnered significant attention in the nanotechnology field due to their distinctive characteristics, including excellent electrical conductivity, chemical stability, and catalytic properties. Various researchers in different fields have been developing the bimetallic nanomaterials using different synthetic approaches as illustrated in Scheme 1, such as the top-down approach and the bottom-up approach.

The synthesis of bimetallic nanomaterials can be achieved using both top-down and bottom-up strategies. The bottom-up strategy involves the construction of nanostructures from atomic or molecular precursors, frequently employing chemical techniques such as reduction with chemical agents. Furthermore, biological sources, such as plant extracts, microorganisms, and enzymes, are being increasingly utilized for green synthesis, providing environmentally sustainable alternatives. These biological techniques utilize natural reducing and capping agents to promote the formation of nanoparticles [4, 5].

The top-down approach involves physical methods for the synthesis of nanomaterials, which often require costly techniques, high pressures and temperatures, and substantial space for equipment. On the other hand, the bottom-up approach utilizes chemical and biological methods for nanoparticle synthesis [4, 5]. While chemical methods can be effective, they often involve hazardous toxic substances that pose risks to both the handler and the environment. Furthermore, researchers have indicated that some of these toxic chemicals may persist in the resulting nanomaterials, potentially leading to safety concerns in medical applications. Nanomaterials synthesized through physical and chemical techniques present distinct advantages and disadvantages. Many of these methods necessitate high temperatures, complex procedures, toxic reagents, specialized equipment, and external additives. The reactions often produce significant waste, and the incorporation of chemical agents can be both costly and labor-intensive. Furthermore, the use of toxic chemicals in these processes poses risks to both the environment and the individuals involved [6, 7]. Traditional approaches to developing bimetallic oxide nanomaterials typically rely on high-temperature methods and hazardous substances, which can be both energy-consuming and harmful to the environment. Due to environmental concerns and the demand for sustainable practices, green synthesis methods have emerged as promising alternatives. These approaches focus on minimizing or eliminating toxic substances and reducing the ecological footprint of material production. Green synthesis involves the development and implementation of chemical processes that are environmentally friendly, energy-efficient, and economically viable, aiming for sustainable production while decreasing the reliance on harmful chemicals, lowering waste generation, and conserving energy.

### 1.3. Green Synthesis of Bimetallic Nanomaterials (Biological Method)

The green synthetic method, often referred to as the sustainable method, was established to enhance the economic viability of chemical production while promoting environmental safety by removing the risks associated with chemical feedstocks, solvents, reagents, and end products [41, 42]. This approach focuses on designing and manufacturing chemical products and processes that are economically competitive while observing the highest standards of pollution prevention by addressing pollution at its source. The green method has gained significant interest among researchers, chemists, and industries engaged in innovative chemical research and applications. Green chemistry presents an opportunity to develop creative solutions to chemical challenges and integrate sustainability into molecular design [43, 44]. Chemists are empowered to create products and processes that minimize their impact on human health and the environment, thereby establishing sustainable chemical building blocks for various materials and products in society. This methodology has been implemented across a diversity of industrial and consumer products, such as paints, dyes, fertilizers, pesticides, plastics, pharmaceuticals, electronics, dry cleaning, energy production, and water treatment. In the context of nanomaterial synthesis, the green approach utilizes plant extracts, microorganisms, bacteria, fungi, algae, and yeast as both reducing and capping agents, particularly for biomedical applications. This method is time-efficient, eliminates the need for intermediates, and does not require expensive precursors or specialized equipment [7, 8]. Nanomaterials produced through green synthesis techniques offer enhanced versatility for applications in biomedical fields, sensors, water splitting, electrochemistry, and various other areas within nanoscience and nanotechnology. Unlike traditional chemical and physical methods, which often involve hazardous and costly chemical reactions, green synthesis minimizes these risks and complexities, thereby broadening the scope of potential applications. This environmentally friendly approach to nanomaterial development is not only safer but also more efficient in terms of time and resource utilization [7–10, 44, 45].

### 1.4. Mechanistic Perspective on Plant-Extract-Mediated Synthesis

The green synthesis of metal nanoparticles utilizing plant extracts presents a sustainable, cost-efficient, and eco-friendly method, offering significant benefits compared to conventional chemical techniques. This biosynthetic approach is noted for its stability, safety for human health, and the removal of toxic substances, making it more suitable for uses that demand biocompatibility and environmental safety. Plant materials, including leaves, fruits, roots, stems, and seeds, are extracted in aqueous solutions, generally employing distilled water, to obtain phytochemicals such as flavonoids, phenolics, alkaloids, and terpenoids. These bioactive substances function in two capacities: as reducing agents that convert metal ions or oxides into zero-valent nanoparticles, and as stabilizers or capping agents through bio-capping, which is facilitated by carboxylic and phenolic acids [19, 26, 27, 32, 46–48].

The reduction process involves electron donation from functional groups such as hydroxyl and carbonyl groups within phytochemicals, which transfer electrons to metal ions, promoting nucleation, the initial formation of seed clusters essential for nanoparticle growth. As nucleation proceeds, these bioactive molecules adsorb selectively onto specific crystallographic facets due to their functional groups. This facet-specific adsorption influences anisotropic growth, allowing control over nanoparticle morphology, resulting in various shapes such as spheres, rods, or plates. For example, phenolic acids can preferentially bind to certain crystal planes, modulating surface energy and directing shape-controlled synthesis [32, 46–48].

Postnucleation, phytochemicals act as capping agents by stabilizing nanoparticles and preventing uncontrolled growth and agglomeration. Their adsorption impacts surface roughness, porosity, dispersity, and defect states, which are crucial for the functional properties of the nanomaterials, including catalytic activity. During subsequent thermal treatments like calcination or annealing, residual phytochemicals influence phase formation and crystallinity by mediating atom mobility and defect incorporation, thereby determining phase purity, surface chemistry, and electronic properties [32, 46–48].

**Figure 1:**
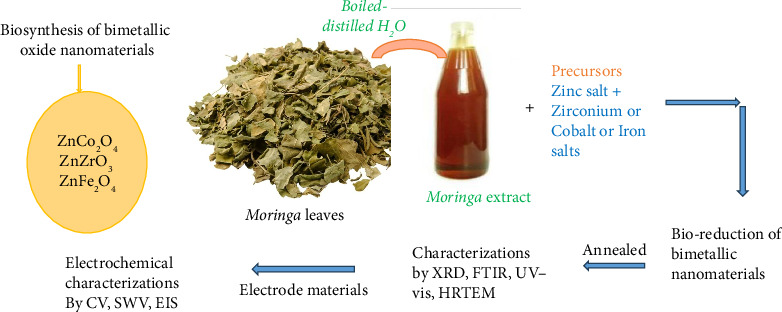
The green synthesis of bimetallic nanomaterials using *Moringa oleifera* plant extract [32, 49, 50].

**Figure 2:**
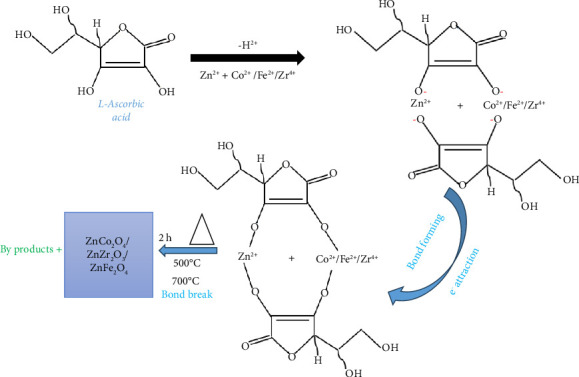
Proposed mechanism toward the formation of the spinel cubic of bimetallic nanomaterial [32, 49, 50].

In specific cases such as the synthesis of bimetallic nanomaterials like ZnCo_2_O_4_, ZnO–ZrO_2_, and ZnFe_2_O_4_ using *M. oleifera* extract, phytochemicals such as ascorbic acid, polyphenols, and flavonoids form complexes with metal ions, guiding nucleation, and growth processes. In this approach, the precursors were completely dissolved by stirring without additional heat. The mixture was dried after 18 h and kept covered with foil at room temperature. The product was treated at temperatures between 500°C and 700°C for 2 h. In Figure 1, we provide the synthesis of bimetallic nanomaterials through a biological compound of *M. oleifera* extract. The proposed mechanism for the green synthesis of the bimetallic nanomaterials (ZnCo_2_O_4_, ZnO–ZrO_2_, and ZnFe_2_O_4_) is depicted in Figure 2 [32, 49, 50]. The phytochemicals present in the plant extract can act as reducing agents for converting the metal precursors to metal nanoparticles. Briefly, the precursors dissociate into metal ions (cations) when in solution, and biological compounds are able to undergo oxidation via free radicals, that is., L-ascorbic acid to L-dehydro ascorbic acid. The complex of metal-ascorbic acid was formed due to the attraction of electrons between the anions of L-hydro ascorbic acid and the metal cations. The metal complex undergoes annealing to form a pure product of bimetallic nanomaterials [32, 49, 50]. These interactions are confirmed through analytical techniques like X-ray diffraction (XRD) and energy-dispersive X-ray spectroscopy (EDX), which verify phase purity. Electron microscopy further reveals that the morphology of the nanomaterials is modulated by the biomolecular interactions, enabling the tailoring of nanostructures for desired applications [32, 49, 50].

### 1.5. Characterization Techniques

To confirm the successful synthesis and elucidate the structural and morphological features of the binary-metal oxide nanomaterials, various characterization techniques are employed:

Characterization techniques such as XRD, scanning electron microscopy (SEM), transmission electron microscopy (TEM), Fourier transform infrared spectroscopy (FTIR), and UV–visible spectroscopy (UV–vis) are essential for confirming the successful synthesis and analyzing the structural and morphological features of binary-metal oxide nanomaterials. XRD identifies crystalline phases and estimates crystallite size, while SEM examines surface morphology and particle distribution. TEM provides detailed insights into nanoparticle size, shape, and crystallinity at high resolution. FTIR detects functional groups involved in reduction and stabilization, confirming oxide formation through characteristic metal–oxygen peaks. UV–vis monitors optical properties and surface plasmon resonance, revealing size-dependent electronic transitions and interactions with phytochemicals, thus offering a comprehensive understanding of the nanomaterials' structural and functional attributes.

## 2. Applications of Plant Extract-Based Binary-Metal Oxide Nanomaterials

The environmentally friendly synthesis of binary-metal oxide nanomaterials using natural extracts from plants has attracted considerable interest due to its sustainable approach and the distinctive physicochemical characteristics of the resulting nanomaterials. These characteristics, including high surface area, reactivity, and catalytic activity, make them highly suitable for a variety of applications in the environmental, biomedical, and energy sectors, thereby promoting sustainable development and innovative technological advancements. Here some recent studies have explored the production of bimetallic nanostructures such as ZnCo_2_O_4_, ZnO–ZrO_2_, and ZnFe_2_O_4_ and their applications through an environmentally friendly approach using different natural plant extracts, as summarized in Table 1.

### 2.1. Environmental Remediation (Photocatalysis and Catalysis)

Environmental contaminants resulting from industrial discharges, agricultural runoff, and urban waste present significant risk to ecosystems and human health. Addressing these issues requires advanced, eco-friendly remediation techniques. Nanotechnology provides promising solutions through the manufacturing of nanomaterials with enhanced surface area, reactivity, and catalytic properties. Binary-metal oxide nanomaterials synthesized from plant extracts, such as ZnCo_2_O_4_, ZnO–ZrO_2_, and ZnFe_2_O_4_, have shown remarkable potential in environmental remediation. These nanomaterials possess exceptional photocatalytic, adsorptive, and catalytic properties that facilitate the removal of heavy metals like lead, arsenic, and cadmium through adsorption and reduction mechanisms. Their photocatalytic efficiency under light exposure also facilitates the degradation of dyes, pharmaceuticals, and organic pollutants, resulting in cleaner water sources and diminished environmental toxicity. Additionally, these nanomaterials act as catalysts in oxidation-reduction reactions, supporting sustainable chemical processes. Their eco-friendly synthesis methods reduce secondary pollution, aligning with sustainable practices.

#### 2.1.1. Photocalysis

Seyed Ali Heidari-Asil et al. (2022) developed magnetically recyclable ZnCo_2_O_4_/Co_3_O_4_ nanocomposites synthesized via a green auto-combustion method using stevia extract. These nanocomposites achieved a photocatalytic degradation efficiency of up to 93.5% for acid violet 7 within 70 min and completely degraded 2-phenol in 18 min under visible light illumination, highlighting their high efficacy in organic pollutant removal [51]. The work of Meky et al. (2023) employed a co-precipitation method using *Pterocladia capillacea* extract as a green capping and reducing agent to synthesize cube-shaped cobalt-doped zinc oxide nanoparticles (Co–ZnO NPs) with varying cobalt contents (5%, 10%, 15%) and demonstrated that 5% Co–ZnO NPs achieved the highest photocatalytic efficiency, degrading 10 mg/L ciprofloxacin (100%) completely within 15 min under visible LED light via a first-order kinetic model (R^2^ = 0.952), thereby highlighting their potential for environmental pollutant remediation [52]. Sirajul Haq et al. (2020) conducted a comprehensive study of *Wedelia biflora-*mediated zinc-cobalt oxide nanocomposite (ZnCo_2_O_4_ NC), their investigation demonstrated the nanocomposite's effective adsorption of Cr(VI) ions following Freundlich and Langmuir models, with thermodynamic analysis indicating an endothermic, 1:1 exchange mechanism, and its high photocatalytic performance in degrading rhodamine 6G (98.92% in 330 min) under simulated solar light, along with good stability and reusability based on desorption and recycling studies [53]. Mohamed A. Hassaan et al. (2024) conducted an experimental study employing hydrothermal synthesis using *P. capillacea extract* to produce Co^2+^-doped ZnO nanoparticles and demonstrated that green 5% Hy-Co–ZnO achieved near-complete (99%) degradation of ciprofloxacin antibiotic under visible LED light within 120 min, following pseudo-first-order kinetics, with sustained performance over three cycles, and optimal degradation efficiency (96.4%) was attained at specific parameters (39.45 ppm CIPF, 60.56 mg catalyst, 177.33 rpm shaking, pH 7.57) [54].

Sirajul Haq et al. (2021) employed a green synthesis route using rubber leaves to produce ZnO–ZrO_2_ heterojunctions, which exhibited remarkable photocatalytic activity, achieving 97.30% degradation of Rhodamine 6G dye, surpassing commercial ZnO nanopowders (86.32%). The heterojunctions also showed strong antioxidant activity, effectively scavenging ABTS free radicals, indicating their promising dual role in environmental and biological applications [55].

Recent studies by Sathisha et al. (2025) further emphasize the efficacy of green-synthesized ZnO/ZrO_2_ nanocomposites using *Butea monosperma* leaf powder as a natural fuel. These nanocomposites achieved 99% degradation of methylene blue dye within 150 min under UV light and reduced hexavalent chromium (Cr(VI)) by 62%, underscoring their environmental remediation potential [56].

Nguyen et al. (2023) employed *Chrysanthemum* floral waste extract for the green synthesis of ZnFe_2_O_4_@ZnO nanocomposites, which demonstrated 94.85% degradation of Congo red dye under solar light at a dye concentration of 5.0 mg/L and catalyst dosage of 0.33 g/L [34].

Nguyen et al. (2022) further reported the green synthesis of ZnFe_2_O_4_ nanoparticles using *Chrysanthemum* flower extract, which effectively adsorbed malachite green dye with a maximum capacity of 121.2 mg/g and achieved 80.4% photocatalytic degradation under solar light, with recyclability over four cycles, emphasizing sustainable wastewater treatment strategies [36].

Vinay Kumar et al. (2023) developed an eco-friendly and cost-effective synthesis method using *B. monosperma* leaf extract through a solution combustion process to produce ZnFe_2_O_4_–ZnO nanocomposites. Their experimental evaluation demonstrated that, under visible light, the heterostructure achieved 95% degradation of Rose Bengal dye. The nanocomposite maintained high reusability over four cycles and exhibited green photoluminescence suitable for forensic applications, indicating its potential as an efficient photocatalyst for organic dye removal [57]. Asri et al. (2023) conducted an experimental study on the photocatalytic removal of humic acid using zinc ferrite nanoparticles loaded with zinc oxide (ZnFe_2_O_4_@ZnO), synthesized via a green method utilizing oleaster tree bark extract. They investigated the effects of pH, nanoparticle dose, and humic acid concentration in a batch reactor. Under optimal conditions, up to 95% degradation of humic acid was achieved within 120 min. The catalyst demonstrated good reusability over five cycles [58].

Sunirmal Saha et al. (2023) employed a green synthesis approach via auto-combustion using *Aloe vera* extract to produce Ag-doped ZnFe_2_O_4_ (ZFO) nanoparticles at 600°C. Their results showed that Ag doping significantly enhanced visible-light-driven photoluminescence and photocatalytic activity, indicating improved charge separation, faster interfacial charge transfer, and higher conductivity. These findings suggest that Ag-doped ZFO nanomaterials are promising for photocatalytic applications and as anti-reflection coatings in photovoltaic devices [59]. R. Meena et al. (2024) developed a green biochar-supported ZnFe_2_O_4_ (BC–ZnFe_2_O_4_) composite catalyst using waste banana peel via a simple calcination process. Photocatalytic experiments demonstrated that BC–ZnFe_2_O_4_ achieved 91.08% degradation of methylene blue within 100 min under visible light, significantly outperforming pure biochar-supported ZnO (75.26%) [37]. Abed et al. (2024) employed a green synthesis approach employing *C. monogyna* leaf extract to produce ZnFe_2_O_4_@Au nanoparticles for degrading pollutants methylene blue and eriochrome black T under UV light, achieving degradation efficiencies of 94.3% and 90.8% within 120 min, respectively, and exhibited notable antibacterial activity with MIC values of 2.5 mg/mL against *Staphylococcus aureus* and *Escherichia coli*, indicating their potential for environmental and biomedical applications [60]. Jayanta Das et al. (2025) synthesized ZnFe_2_O_4_ nanoparticles (CS–ZnFe_2_O_4_ NPs) using a green method with *Camellia sinensis* shoots, the results demonstrated that these nanoparticles exhibited excellent catalytic activity, achieving 89% degradation of the pesticide thiamethoxam within 60 min under solar radiation [61]. Umer Younas et al. (2023) conducted an experimental study on the green synthesis of franklinite (ZnFe_2_O_4_) nanoparticles using extracts of fresh and dried *Coriandrum sativum* leaves; their results demonstrated that the biosynthesized NPs exhibited high catalytic efficiency in degrading eosin yellow (up to 86% in 22 min using dried leaf extract and 90% in 16 min using fresh leaf extract) and significant antioxidant activity [62].

#### 2.1.2. Catalysis

Goudarzi et al. summarized the green synthesis of ZnCo_2_O_4_/ZnO nanoparticles using *D. coccus,* noting their significant cytotoxicity against 4T1 breast cancer cells via oxidative stress and apoptosis pathways, indicating potential biomedical applications alongside environmental remediation [33]. Adaileh et al. (2025) fabricated a polyacrylonitrile polymer composite incorporating Cu–ZnO/ZrO_2_ nanoparticles, capable of removing heavy metals such as Pb(II) and Cd(II), along with pharmaceutical pollutants like sulfamethoxazole (SMX) and ibuprofen. The composite demonstrated high degradation efficiencies of up to 90% for SMX and 88% for ibuprofen within 120 min and maintained about 85% activity over five reuse cycles, signifying its sustainability [63].

The work of Luo et al. (2025) presents a comprehensive study on the preparation, characterization, and catalytic performance of Cu–ZnO–ZrO_2_ (CZZ) catalysts with varying Cu/Zn molar ratios aimed at enhancing CO_2_ hydrogenation to methanol. Their findings suggest that optimizing the molar ratio of Cu/Zn enhances the formation of active alloy and interface sites, leading to improved catalytic performance. The synergistic effects among Cu, ZnO, and ZrO_2_ are critical for the activation and conversion of CO_2_, with oxygen vacancies playing a pivotal role [64].

Sattar H. Abed et al. (2025) synthesized ZnFe_2_O_4_@Au nanoparticles using *C. monogyna* leaf extract, demonstrating high catalytic efficiency in degrading methylene blue and eriochrome black T dyes with efficiencies of 94.3% and 90.8%, respectively, within 120 min. These nanoparticles also exhibited notable antibacterial activity against both Gram-positive and Gram-negative bacteria, with MIC values of 2.5 mg/mL indicating their multifaceted utility in environmental and health sectors [35].

The work of Mandal et al. (2020) demonstrates a green synthesis approach for producing phase-pure polygonal ZnCo_2_O_4_ nanoparticles using extracts from *Punica granatum* peel*, Camellia sinensis*, *M. oleifera*, and green coffee beans, revealing that the *Punica granatum-*assisted sample exhibits superior catalytic degradation (∼84.96%) of Rhodamine B dye, high electrical conductivity (∼8.074 × 10^−5^ Ω^−1^ cm^−1^ with an activation energy of 2.099 eV at 503 K), and effective antibacterial activity against *Bacillus* species, indicating the potential of eco-friendly plant extracts in fabricating functional nanomaterials for catalysis, electronics, and antimicrobial applications [65].

Sampat R. Shingda et al. (2025) reported the green synthesis of ZnFe_2_O_4_@AC nanocomposites using *Careya arborea* leaf extract via a co-precipitation method, with characterization confirming cubic crystalline structure and nanoscale size (18–22 nm), which demonstrated high catalytic efficiency in the room-temperature synthesis of Quinazolin-4(1H)-one derivatives, with 20 mg of catalyst achieving up to 96% yield in ethanol with simple procedures, mild conditions, and high purity [66].

### 2.2. Energy Storage and Conversion, Sensors

Bimetallic nanocomposites are essential in advancing electrochemical technologies, especially in the fields of energy storage and conversion. Their remarkable electrical conductivity, electrochemical stability, and extensive surface area contribute to outstanding performance in batteries, supercapacitors, sensors, and fuel cells. Furthermore, bimetallic nanomaterials demonstrate exceptional catalytic activity for biosensing, sensing of organic pollutants, and glucose detection, indicating potential advancements in medical diagnostics and environmental monitoring. The combination of binary and ternary transition metal oxides significantly boosts electrochemical properties by enhancing conductivity and structural stability. These bimetallic nanocomposites are crucial for the development of sustainable, high-performance energy and sensing devices.

Kiruthika et al. (2022) employed a green sol–gel synthesis method using *Actinidia deliciosa* fruit extract as a reducing agent to produce ZnFe_2_O_4_ spinel nanoparticles aimed at anode application in Li-ion batteries, and electrochemical testing indicated favorable electrical conductivity and activity, suggesting the material's promising potential as an anode in lithium-ion batteries [67].

V. Lakshmi Ranganatha et al. (2020) conducted an experimental study on synthesizing zinc ferrite (ZnFe_2_O_4_) nanoparticles via a cost-effective, eco-friendly method using *Aegle marmelos extract* as a fuel. The study found that the nanoparticles exhibited high photocatalytic efficiency, with a 96% degradation of methylene blue under visible light, demonstrating active reusability; electrochemical properties assessed by cyclic voltammetry and EIS indicated promising electrochemical behavior, and antimicrobial tests against bacteria and fungi indicated significant antimicrobial activity, suggesting that ZnFe_2_O_4_ nanoparticles have potential applications as photocatalysts, electrochemical sensors, and antimicrobial agents [68]. Mahnaz Amiri and Hadi Mahmoudi-Moghaddam (2021) reported the green synthesis of ZnO/ZnCo_2_O_4_ nanocomposite via a hydrothermal method utilizing *G. glabra* extract as a natural reducing and capping agent and demonstrated its application as an electrode modifier for detecting bisphenol A (BPA) through differential pulse voltammetry (DPV), achieving a linear detection range of 0.06–200.0 µM and a detection limit of 0.01 µM, along with high selectivity, stability, and repeatability, effectively detecting BPA in water samples [38]. Bekele et al. (2024) synthesized green Co_2_O_4_, ZnO, (NPs) ZnO/ZnCo_2_O_4_ (NCs) nanocomposites using *R. communis* leaf extract and characterized their structural, optical, and electrochemical properties; their experimental findings demonstrated that these nanocomposites, when used as working electrodes, exhibited enhanced charge transfer, high surface area, and electrocatalytic activity, resulting in improved sensitivity and a low detection limit (91.25 µA−1 µM−1 cm−2) for the electrochemical sensing of SMX, highlighting their potential for selective and stable pollutant detection [39].

This study by S. Jeevitha et al. developed a green synthesis method using papaya leaf extract to produce ZnFe_2_O_4_ nanoparticles. The nanoparticles demonstrated effective properties for sensor applications for detecting acetaminophen, showing a low detection limit of 0.5274 µM and a linear range of 0.1–40 µM. The sensor exhibited high accuracy, reproducibility, stability, and selectivity in real pharmaceutical samples like Zerodol P and Dolo drops. These results highlight the potential of ZnFe_2_O_4_ nanoparticles to improve pharmaceutical detection technologies [69]. Raj et al. (2024) successfully synthesized an Ag–ZnFe_2_O_4_@graphene nanocomposite using *Artocarpus heterophyllus* leaf extract via a hydrothermal method, which produced 20–30 nm ZnFe_2_O_4_ particles with well-distributed Ag dots and graphene sheets; the composite demonstrated complete degradation of methylene blue within 180 min due to enhanced charge separation and reduced electron-hole recombination and exhibited notable electrochemical sensing capabilities for dopamine with a low detection limit of 1.6 μM, highlighting its potential for environmental remediation and biosensing applications as a sustainable nanomaterial fabricated through green synthesis methods [70].

### 2.3. Biomedical Application

Nanomaterials based on plant extract-derived binary-metal oxides have emerged as a promising area in medicine due to their distinctive physicochemical characteristics, biocompatibility, and environmentally friendly synthesis techniques. These nanomaterials utilize the natural reducing and stabilizing elements found in plant extracts to create stable, multifunctional nanostructures that can be customized for a range of biomedical uses. By providing a sustainable and multifunctional platform, they enhance biomedical technology through the facilitation of targeted therapies, imaging, and regenerative medicine. Their eco-friendly synthesis, along with the intrinsic antioxidant and anti-inflammatory properties derived from plant phytochemicals, unlocks the way for new strategies in managing chronic inflammatory conditions and nurturing tissue regeneration, thereby positioning them as adaptable instruments for future advancements in healthcare. Tuama and Alias (2024) detailed the green synthesis of ZnO: ZrO_2_ nanocomposites using plant extracts from *Z. officinale* and *Syzygium aromaticum*, demonstrating significant anticancer activity against colon cancer cell lines (HCT-116 and LoVo), inducing apoptosis and cell death. These findings suggest that environmentally friendly synthesized nanocomposites hold promising potential for medical applications, especially in combating microbial resistance and cancer treatment [71]. The author AM Korotkova et al. (2024) conducted an experimental study on the green synthesis of zinc ferrite (ZnFe_2_O_4_) nanoparticles using *Petroselinum crispum*, characterizing the particles with SEM techniques; they evaluated the biological effects on wheat (*Triticum vulgare* L.) grown in hydroponic medium with varying nanoparticle concentrations (10−5 to 10−1 M), observing that acidi-synthesized particles significantly reduced seed germination and cell viability, especially at higher concentrations, whereas alkaline-synthesized particles had a lesser inhibitory effect and even slightly promoted germination and cell viability at lower concentrations [72].

Imraish et al. (2021) reported that using an eco-friendly green biosynthesis method involving *B. carteri* resin extract, zinc acetate, and iron chloride, they successfully synthesized cubic ZnFe2O4 bimetallic nanoparticles averaging 10.54 nm in size, characterized through various analytical techniques, and demonstrated their selective potent anticancer activity against K562 and MDA-MB-231 cell lines with IC50 values of 4.53 μM and 4.19 μM respectively via MTT cytotoxicity assays, indicating their potential as environmentally friendly, low-cost chemotherapeutic agents with minimal effects on normal fibroblasts, though further in vivo studies are necessary to confirm their applicability [73].

Imraish et al. (2021) conducted an experimental study employing green synthesis methods using *B. carteri* resin extract to produce bimetallic ZnFe_2_O_4_ and CrFe_2_O_4_; their findings demonstrated that both nanoparticles exhibited moderate hydrogen peroxide scavenging activity (IC50 ∼87.5 and 146.4 μg/mL), potent nitric oxide scavenging (IC50 ∼4.01 μg/mL), and significant anti-inflammatory effects through dose-dependent suppression of proinflammatory cytokines (IL-1β, IL-6, TNF-α) at the mRNA and protein levels, indicating their promising potential for biomedical applications such as anti-inflammatory and antioxidant agents [40]. Supriya Gumma et al. (2024) conducted an experimental study utilizing a novel phytochemical synthesis method with *Catharanthus roseus* leaf extract to produce ZnCo_2_O_4_ and Pd@ZnCo_2_O_4_ nanoparticles; their photocatalytic activity was evaluated through the degradation of brilliant blue dye under 40 min, achieving 42% and 97% degradation, respectively, demonstrating an eco-friendly, effective approach for dye photodegradation, with additional evaluation of their antibacterial and antioxidant properties indicating promising biological applications [74]. Krishnan and colleagues (2021) conducted a study employing a facile hydrothermal green synthesis method using orange peel extract as a natural surfactant to produce ZnFe_2_O_4_/reduced graphene oxide nanohybrids (G–ZnFe_2_O_4_/rGO NHs), which were thoroughly characterized and evaluated for photocatalytic degradation of methylene blue, showing a maximum efficiency of 92.4% under visible light; additionally, these nanohybrids demonstrated significant antibacterial activity against *E. coli* with a bacterial survival rate of approximately 29.43% after 40 min of irradiation, and exhibited notable cytotoxic effects on A549 lung cancer cells with an IC50 of 249.9 μg/mL, indicating their potential for environmental remediation and biomedical applications, as demonstrated through experimental synthesis, characterization, photocatalytic testing, antibacterial assays, and in vitro cytotoxicity evaluations [75].

## 3. Compared Green Synthesis to Traditional Physical and Chemical Synthesis Methods

In comparison to conventional physical and chemical synthesis techniques, green synthesis, particularly through the use of plant extracts, presents notable benefits regarding sustainability, environmental friendliness, and cost-effectiveness.

In green synthesis, biological pathways are employed, utilizing microorganisms, plant extracts, or natural phytochemicals that serve as reducing and stabilizing agents. These techniques function under mild conditions, eliminate the necessity for toxic substances, and utilize renewable resources, thus minimizing hazardous waste and energy usage. Approaches based on plants are designed to reduce environmental impact while improving the stability, uniformity, and functional performance of the resulting nanomaterials. They can generate nanostructures with controlled morphology, enhanced photocatalytic activity, and surface modifications that increase their applicability in fields such as medicine, environmental remediation, and energy storage, as evidenced by various research studies [46–48].

Traditional methods, including sol–gel, hydrothermal, chemical vapor deposition, and physical techniques like pulsed laser ablation, frequently require high temperatures, involve hazardous chemicals, entail complex procedures, and produce significant waste, which raises environmental and safety issues. Although these methods can yield high-purity, well-controlled nanomaterials, their processes are often energy-intensive, expensive, and less environmentally friendly [4–7].

Despite ongoing challenges related to scaling up, reproducibility, and standardization, green synthesis is increasingly recognized as a promising, sustainable alternative that aligns with global objectives for environmentally responsible production of nanomaterials.

## 4. Conclusion and Future Prospects

This review highlights the considerable potential of green synthesis using plant extracts to produce binary-metal oxide nanomaterials, aligning with the principles of sustainable chemistry. This approach provides a cost-effective and nontoxic alternative to traditional methods, resulting in functional nanostructures that can be utilized in environmental, biomedical, and industrial applications. Despite significant advancements, challenges persist, including variability in phytochemical composition, the ability to control particle size and morphology, and issues related to scalability. Future research should focus on standardizing extraction methods, gaining a mechanistic understanding of bio-reduction processes, and optimizing processes to enable industrial application. Improvements in characterization techniques and the integration of other sustainable technologies are expected to enhance the functional properties and expand the application range of these nanomaterials. The use of natural extracts is likely to advance green manufacturing practices, minimize chemical waste, and promote innovations in energy storage, environmental remediation, and biomedicine. Collaborations across disciplines will be critical for the development of next-generation, eco-friendly nanomaterials that meet the requirements of a sustainable future.

## Figures and Tables

**Table 1:** Summary of binary metal oxide nanomaterials synthesized using plant natural extracts for different applications.

Sector	Types of binary	Methods synthesis	Application	Results % Degradation	Year	Ref.
Biomedical	ZnCo_2_O_4_/ZnO	*Dactylopius coccus*	4T1 breast cancer cells			[33]
Environmental	ZnFe_2_O_4_@ZnO	*Chrysanthemum floral* waste extract	Congo red dye	94.85%	2023	[34]
Environmental and biomedical	ZnFe_2_O_4_@Au	*Crataegus monogyna* leaf extract	Methylene blue and eriochrome black T dyes have antibacterial activity	94.3% and 90.8%,	2025	[35]
Environmental	ZnFe_2_O_4_	*Chrysanthemum* flower extract	Malachite green dye	80.4%	2022	[36]
Environmental	BC–ZnFe_2_O_4_	Waste banana peel	Methylene blue	91.08%.	2024	[37]
Electrochemical	ZnO/ZnCo_2_O_4_	*Ricinus communis* leaf extract	Sulfamethoxazole (SMX),	Detection limit (91.25 µA−1 µM−1 cm−2)	2024	[39]
Biomedical and antioxidant	ZnFe_2_O_4_ and CrFe_2_O_4_	*Boswellia carteri* resin extract	Hydrogen peroxide scavenging activity potent nitric oxide scavenging	(IC50 ∼87.5 and 146.4 μg/mL), (IC50 ∼4.01 μg/mL)	2021	[40]
Environmental	ZnCo_2_O_4_/Co_3_O_4_	*Stevia* extract	Acid violet 7	93.5%		[51]
			2-Phenol			
Environmental	Co–ZnONPs	*Pterocladia capillacea* extract	Ciprofloxacin	100%	2023	[52]
Environmental	(ZnCo_2_O_4_ NC)	*Wedelia biflora—*studies	Adsorption of Cr(VI) ions degrading rhodamine 6G	98.92%	2020	[53]
Environmental	Co^2+^-doped ZnO.	*P. capillacea* extract	Ciprofloxacin antibiotic	99%	2024	[54]
Environmental and biomedical	ZnO–ZrO_2_	Rubber leaves	Degradation of rhodamine 6G dye antioxidant activity	97.30%	2021	[55]
Environmental	ZnO/ZrO_2_	*Butea monosperma* leaf powder	Methylene blue and reduced hexavalent chromium	99%	2025	[56]
Environmental	ZnFe_2_O_4_–ZnO nanocomposites.	*Butea monosperma* leaf extract	Degradation of rose Bengal dye	95%	2023	[57]
Environmental	ZnFe_2_O_4_@ZnO	*Oleaster* tree bark extract	Humic acid	95%	2023	[58]
Photovoltaic	Ag-doped ZnFe_2_O_4_	*Aloe vera* extract	Coatings in photovoltaic devices		2023	[59]
Environmental	CS–ZnFe_2_O_4_ NPs	*Camellia sinensis* shoots	Pesticide thiamethoxam	89%	2025	[61]
Environmental	ZnFe_2_O_4_	Extracts of fresh and dried *Coriandrum sativum* leaves	Eosin yellow	86%	2023	[62]
				90%		
Environmental	Cu–ZnO/ZrO_2_		Sulfamethoxazole and ibuprofen	90% and 88%	2025	[63]
Environmental and biomedical	ZnCo_2_O_4_	*Punica granatum* peel*, Camellia sinensis, Moringa oleifera,* and green coffee beans	Rhodamine B dye, antibacterial activity	84.96%	2020	[65]
Catalysis	ZnFe_2_O_4_@AC	*Careya arborea* leaf extract	Catalytic efficiency Quinazolin-4(1H)-one derivatives		2025	[66]
Electrochemical	ZnFe_2_O_4_	*Actinidia deliciosa* fruit extract	Li-ion batteries.		2022	[67]
Environmental electrochemical	ZnFe_2_O_4_	*Aegle marmelos* extract	Methylene blue electrochemical antimicrobial	96%	2020	[68]
Electrochemical	ZnO/ZnCo_2_O_4_	*Glycyrrhiza glabra* extract	Bisphenol A (BPA)	Detection limit of 0.01 µM,.	2021	[38]
Electrochemical	ZnFe_2_O_4_	Papaya leaf extract	Zerodol P and Dolo drops	Detection limit of 0.5274 µM		[69]
Environmental and electrochemical	Ag–ZnFe_2_O_4_@graphen	*Artocarpus heterophyllus* leaf extract	Methylene blue and Dopamin	Detection limit of 1.6 μM,	2024	[70]
Biomedical	ZnO: ZrO_2_	Plant extracts from *Z. officinale* and *S. aromaticum*	Colon cancer cell lines (HCT-116 and LoVo)		2024	[71]
Environmental	ZnFe_2_O_4_	*Petroselinum crispum*	*Triticum vulgare* L.		2024	[72]
Biomedical	ZnFe_2_O_4_	*Boswellia carteri* resin extract	K562 and MDA-MB-231	4.53 μM and 4.19 μM	2021	[73]
Environmental and biomedical	ZnCo_2_O_4_ and Pd@ZnCo_2_O_4_	*Catharanthus roseus* leaf extract	Brilliant blue dye	42%	2024	[74]
			Antibacterial and antioxidant	97%		
Environmental and biomedical	G–ZnFe_2_O_4_/rGO NHs	Orange peel extract	Methylene blue,	92.4%	2021	[75]
			*Escherichia coli*	29.43%		

## Data Availability

Data sharing is not applicable to this article, as no datasets were generated or analyzed during the current study.
